# Surgical Safety of Uniportal versus Multiportal VATS Segmentectomy during Institutional Adoption: Differential Impact by Lobar Location

**DOI:** 10.5761/atcs.oa.26-00042

**Published:** 2026-06-20

**Authors:** Hiroshi Yabuki, Shingo Miyabe, Fumiko Tomiyama, Masafumi Noda

**Affiliations:** Department of Thoracic Surgery, Miyagi Cancer Center, Natori, Miyagi, Japan

**Keywords:** segmentectomy, uniportal video-assisted thoracoscopic surgery, multiportal video-assisted thoracoscopic surgery, surgical safety, postoperative complication

## Abstract

**Purpose:**

This study aimed to compare surgical safety outcomes of uniportal and multiportal video-assisted thoracoscopic surgery (uVATS and mVATS) segmentectomy during institutional adoption, focusing on differences according to lobar location.

**Methods:**

We retrospectively analyzed 117 patients who underwent thoracoscopic segmentectomy between 2019 and 2024 (uVATS, 49; mVATS, 68). Outcomes included intraoperative pulmonary vessel injury and major postoperative complications, along with operative time, blood loss, and length of hospital stay. Inverse probability of treatment weighting was applied, and analyses were performed overall and stratified by lobe.

**Results:**

Pulmonary vessel injury occurred in 12 patients (10.3%), and grade ≥III complications in 10 (8.5%). After adjustment, uVATS showed a higher incidence of vascular injury than mVATS overall (22.8% vs. 5.5%; p = 0.006). No statistically significant differences were observed in upper-lobe segmentectomy. In lower-lobe segmentectomy, uVATS showed higher rates of vascular injury (44.8% vs. 2.2%; p <0.001) and grade ≥III complications (18.8% vs. 0%; p = 0.005).

**Conclusions:**

No statistically significant differences were observed between uVATS and mVATS in upper-lobe segmentectomy. However, during the institutional adoption phase, careful consideration is warranted when performing lower-lobe segmentectomy with uVATS due to a higher incidence of complications related to surgical safety.

## Introduction

The JCOG0802/WJOG4607L^1)^ and CALGB140503^2)^ trials demonstrated that sublobar resection (segmentectomy or wedge resection) is non-inferior, and possibly superior, to lobectomy. Consequently, segmentectomy has gained acceptance as a curative option for early-stage lung cancer, and approximately 40% of primary lung cancer resections in Japan are now performed as sublobar resections.^3)^

Compared with lobectomy, segmentectomy is technically more demanding because it requires accurate identification of intersegmental planes and precise dissection of pulmonary vessels and bronchi.^4)^ The degree of technical difficulty varies according to lobar location, as anatomical characteristics and surgical exposure differ between upper- and lower-lobe segmentectomies.^5,6)^

Video-assisted thoracoscopic surgery (VATS) has become a standard minimally invasive approach for lung resection. In addition to conventional multiportal VATS (mVATS), uniportal VATS (uVATS) has been introduced, enabling procedures through a single small incision.^7)^ Previous studies have reported favorable perioperative outcomes with uVATS, including shorter hospital stays and reduced postoperative pain, compared with mVATS.^8)^ Other studies have shown comparable operative time, blood loss, and postoperative complication rates between the 2 approaches for segmentectomy.^9,10)^ A systematic review also suggested lower postoperative complication rates with uVATS for both segmentectomy and lobectomy.^11)^ However, uVATS requires advanced technical proficiency.^12)^ Furthermore, lower-lobe segmentectomy is generally considered more challenging than upper-lobe segmentectomy because of deeper hilar structures, greater anatomic variation, and more complex intersegmental planes.^5,6)^

Most previous studies comparing uVATS and mVATS have evaluated segmentectomy as a single category,^8,10)^ without stratified analysis according to lobar location. We hypothesized that, during the phase of institutional adoption of VATS segmentectomy, the surgical safety profile of uVATS might differ from that of mVATS according to lobar location, particularly in lower-lobe resections. Therefore, this study aimed to compare surgical safety outcomes of uVATS and mVATS segmentectomy according to lobar location, with particular focus on differential impacts between upper- and lower-lobe resections during the phase of institutional adoption of VATS segmentectomy. This study was designed as an exploratory analysis of surgical safety during the institutional adoption of uVATS compared with mVATS.

## Materials and Methods

### Patients

We retrospectively reviewed consecutive patients who underwent thoracoscopic pulmonary segmentectomy at Miyagi Cancer Center between January 2019 and December 2024. Patients who underwent simultaneous bilateral surgery, combined segmentectomy and lobectomy, or robot-assisted surgery were excluded.

Segmentectomies were classified as simple (superior segment [S6], basal segmentectomy, left upper-division, or lingular segmentectomy) or complex (all other resections). Collected variables included age, sex, body mass index (BMI), smoking history, performance status (PS), pulmonary function, comorbidities, year of surgery, histopathologic diagnosis, type of segmentectomy, and surgical approach (uVATS or mVATS). Comorbidities were assessed using the Charlson Comorbidity Index (CCI).^13)^

### Surgical procedures

Video-assisted thoracoscopic segmentectomy was introduced at our institution in 2018. mVATS was performed using either a 3- to 4-port confront upside-down technique until March 2022^14)^ or a 3-port look-up technique thereafter. uVATS segmentectomy was introduced in 2019. For upper-lobe segmentectomy, a 3–4 cm incision was made at the fifth intercostal space along the midaxillary line, whereas for lower-lobe segmentectomy, the incision was placed at the sixth intercostal space. The camera was positioned in the facing orientation. Pulmonary vessels were managed according to their size, using energy devices alone for very small vessels, a combination of clips or ligation with energy devices for intermediate-sized vessels, and endoscopic staplers for larger vessels. The choice of technique was based on intraoperative judgment by the operating surgeon. Intersegmental planes were identified using indocyanine green fluorescence or the inflation–deflation method and were divided with endoscopic staplers. The choice of surgical approach was determined by the operating surgeon. No specific devices or techniques unique to the uniportal approach were routinely applied.

### Surgeon experience

Surgeon experience was defined in an approach-specific manner. For each surgeon, cases were categorized as being performed during the early phase of adoption if they occurred within the first 10 segmentectomies using the respective approach (mVATS or uVATS). This definition was intended to distinguish the very early adoption period and does not represent completion of the learning curve.

### Postoperative management and assessment of complications

Postoperative care was provided by a dedicated thoracic surgery team. An intercostal nerve block with ropivacaine was administered prior to chest closure in all patients, followed by scheduled oral non-opioid analgesics. Epidural anesthesia was not routinely used and was not applied in any cases in this cohort. Chest drains were removed when there was no air leak, drainage was neither bloody nor chylous, and the daily drainage volume was ≤200 mL. Chemical pleurodesis was performed if spontaneous closure of an air leak was not anticipated or if air leakage persisted for more than 5 days. Postoperative complications were graded according to the Clavien–Dindo classification.^15)^

### Surgical outcomes

Perioperative outcomes included operative time, blood loss, intraoperative pulmonary vessel injury, duration of chest drainage, postoperative hospital stay, and Clavien–Dindo grade ≥III complications. Pulmonary vessel injury was defined as unexpected intraoperative bleeding from the pulmonary artery or vein requiring hemostatic intervention, such as compression, application of hemostatic materials, or suture repair, reflecting clinical judgment rather than a quantitative blood loss. Pulmonary vessel injury was assessed retrospectively based on operative records and intraoperative findings documented in surgical reports.

The primary endpoints were intraoperative pulmonary vessel injury and Clavien–Dindo grade ≥III complications. Secondary endpoints included operative time, blood loss, duration of chest drainage, and postoperative hospital stay. Analyses were performed for all segmentectomies and separately for upper- and lower-lobe segmentectomies.

### Statistical analysis

All analyses were performed using JMP Student Edition 18 (SAS Institute Inc., Cary, NC, USA). To minimize bias, standardized inverse probability of treatment weighting (IPTW) was applied to compare mVATS and uVATS. Propensity scores for IPTW were estimated using covariates appropriate for each analysis. For all segmentectomy cases, covariates included age, sex, PS, BMI, smoking history (≥10 pack-years), percentage of forced expiratory volume at one second (%FEV_1_), CCI, pathological diagnosis (primary lung cancer, metastatic tumor, or benign), simple versus complex segmentectomy, upper versus lower lobe, year of surgery, and surgeon experience. For upper-lobe segmentectomy, covariates included age, sex, PS, %FEV_1_, smoking history, CCI, simple versus complex segmentectomy, and the year of surgery. For lower-lobe segmentectomy, covariates included sex, PS, BMI, %FEV_1_, smoking history, CCI, year of surgery, and surgeon experience. Covariate balance after IPTW was assessed using standardized mean differences (SMD), with SMD <0.1 considered well balanced and 0.1 ≤SMD <0.2 deemed clinically acceptable given sample size constraints. Weighted linear regression was used for continuous variables, and weighted logistic regression was used for categorical variables. Statistical significance was assessed using likelihood ratio tests. A p-value <0.05 was considered statistically significant.

## Results

### Patient enrollment

During the study period, 137 patients underwent thoracoscopic pulmonary segmentectomy. After excluding 18 patients who underwent robot-assisted surgery and 2 who underwent thoracotomy, 117 patients who underwent segmentectomy via mVATS or uVATS were included in the analysis.

### Patient’s characteristics and surgeon’s experiences

Baseline characteristics are summarized in **Table 1**. Forty-nine patients underwent uVATS and 68 underwent mVATS. Eight surgeons performed segmentectomies during the study period, including 4 surgeons for uVATS and 8 for mVATS. The surgeon’s experiences by lobe and approach during the study period were shown in **Table 2**. Cases were distributed among multiple surgeons, and the number of cases per surgeon was relatively small, reflecting the institutional adoption phase. Systematic mediastinal lymph node dissection was rarely performed in this cohort, with only 1 patient undergoing mediastinal dissection. Most cases involved no mediastinal dissection.

**Table 1:** Patient backgrounds of overall cases before and after standardized inverse probability of treatment weighting

	uVATS	mVATS	p-Value	SMD	IPTW weighted		
					uVATS	mVATS	SMD
n	49	68			49.5	67.6	
Age	70.4 ± 11.0	69.4 ± 9.1	0.612	0.099	70.1 ± 11.1	69.9 ± 8.8	0.020
Sex (male %)	55.1	45.6	0.310	0.190	50.5	50.2	0.006
BMI	23.7 ± 3.8	23.1 ± 3.8	0.384	0.158	23.1 ± 3.7	23.2 ± 3.7	0.027
Smoking history							
10 pack-year or more (%)	46.9	48.5	0.384	0.032	49.9	49.0	0.018
Performance status							
PS ≥1 (%)	14.3	11.8	0.689	0.074	12.8	12.4	0.012
Pulmonary function							
FEV_1_%	74.2 ± 9.1	75.3 ± 9.3	0.541	0.120	74.7 ± 8.5	74.9 ± 9.1	0.023
%FEV_1_	96.8 ± 23.5	96.5 ± 18.9	0.946	0.014	96.7 ± 22.5	95.7 ± 18.9	0.048
%VC	99.5 ± 17.2	98.7 ± 16.1	0.806	0.048	98.7 ± 17.4	98.6 ± 16.1	0.006
Charlson Comorbidity Index			0.301				
0–2	10.2	20.6		0.288	16.3	16.4	0.003
3–5	63.3	54.4		0.181	55.9	56.7	0.016
≥6	26.5	25.0		0.034	27.9	26.9	0.022
Year of surgery			0.885				
2019–2021 (%)	46.9	45.6		0.026	45.8	45.3	0.010
2022–2024 (%)	53.1	54.4		0.026	54.6	54.8	0.004
Diagnosis			0.598				
Primary lung cancer (%)	59.2	63.2		0.082	61.9	61.6	0.006
Metastatic tumor (%)	32.7	25.0		0.170	27.0	27.9	0.020
Benign (%)	8.2	11.8		0.120	11.1	10.5	0.019
Simple/complex segmentectomy							
Complex segmentectomy (%)	63.2	55.9	0.422	0.108	59.8	58.7	0.022
Upper-lobe/lower-lobe segmentectomy							
Upper lobe segmentectomy (%)	55.1	51.5	0.698	0.072	52.2	53.8	0.032
Experienced surgeon (≥10 cases) (%)	34.7	41.2	0.476	0.134	37.0	38.0	0.021

BMI, body mass index; IPTW, inverse probability of treatment weighting; mVATS, multiportal video-assisted thoracoscopic surgery; SMD, standardized mean differences; uVATS, uniportal video-assisted thoracoscopic surgery; %FEV1, percentage of forced expiratory volume at one second; PS, performance status; VC, vital capacity

**Table 2:** Number of surgeons and number of cases per surgeon by lobe and approach during the study period

	uVATS	mVATS
Number of surgeons		
Upper lobe	4	8
Lower lobe	4	7
Number of cases per surgeon, median (range)		
Upper lobe	8 (2–10)	4 (2–10)
Lower lobe	4.5 (2–9)	3.5 (2–9)

uVATS, uniportal video-assisted thoracoscopic surgery; mVATS, multiportal video-assisted thoracoscopic surgery

After IPTW, baseline covariates were generally well balanced between groups (SMD <0.1). The distribution of resected segments is shown in **Supplementary Table 1**.

### Subgroup analysis by lobe

Among the 117 patients, 63 underwent upper-lobe segmentectomy and 54 underwent lower-lobe segmentectomy (**Tables 3** and **4**). In the upper-lobe cohort, 28 patients underwent uVATS and 35 underwent mVATS, whereas in the lower-lobe cohort, 21 underwent uVATS and 33 underwent mVATS. After IPTW, the percentage of vital capacity, CCI, and the proportion of benign disease in the upper-lobe cohort had SMD values between 0.1 and 0.2, while all other variables were well balanced (SMD <0.1). In the lower-lobe cohort, age, %FEV_1_, and the proportion of primary lung cancer had SMD values between 0.1 and 0.2, which was considered acceptable; however, the proportion of metastatic tumors showed an imbalance (SMD = 0.217 >0.2). Other variables were generally well balanced.

**Table 3:** Patient backgrounds of upper-lobe segmentectomy ceases before and after standardized inverse probability of treatment weighting

	uVATS	mVATS	p-Value	SMD	IPTW weighted		
					uVATS	mVATS	SMD
n	28	35			26.6	34.8	
Age	71.4 ± 9.6	68.7 ± 11.3	0.326	0.258	70.7 ± 9.6	69.1 ± 11.0	0.155
Sex (male %)	48.1	54.3	0.849	0.124	60.2	57.9	0.047
BMI	23.5 ± 3.7	23.4 ± 3.9	0.892	0.026	23.4 ± 3.4	23.5 ± 3.7	0.028
Smoking history							
10 pack-year or more (%)	40.7	57.1	0.200	0.328	53.0	51.8	0.024
Performance status							
PS ≥1 (%)	7.4	11.4	0.595	0.137	6.3	8.6	0.088
Pulmonary function							
FEV_1_%	74.9 ± 8.2	73.9 ± 8.7	0.645	0.118	74.2 ± 8.7	73.8 ± 8.5	0.047
%FEV_1_	100.1 ± 22.7	93.0 ± 19.4	0.186	0.336	94.8 ± 21.0	94.4 ± 19.1	0.020
%VC	101.7 ± 17.4	98.1 ± 16.1	0.409	0.215	97.7 ± 15.3	99.8 ± 15.2	0.138
Charlson Comorbidity Index			0.415				
0–2	11.1	22.9		0.304	12.8	17.3	0.126
3–5	66.7	62.9		0.080	69.7	62.9	0.144
≥6	22.2	14.3		0.205	17.5	19.8	0.059
Year of surgery			0.678				
2019–2021 (%)	48.1	42.9		0.104	40.9	40.6	0.006
2022–2024 (%)	51.9	57.1		0.104	59.1	59.4	0.006
Diagnosis			0.634				
Primary lung cancer (%)	63.0	68.6		0.118	66.6	63.3	0.069
Metastatc tumor (%)	29.6	20.0		0.222	27.0	25.7	0.030
Benign (%)	7.4	11.4		0.137	6.5	11.0	0.159
Simple/complex segmentectomy							
Complex segmentectomy (%)	75.0	62.9	0.301	0.415	71.2	70.1	0.024
Experienced surgeon (≥10 cases) (%)	44.4	45.7	0.921	0.026	37.2	41.3	0.084

BMI, body mass index; IPTW, inverse probability of treatment weighting; mVATS, multiportal video-assisted thoracoscopic surgery; SMD, standardized mean differences; uVATS, uniportal video-assisted thoracoscopic surgery; %FEV1, percentage of forced expiratory volume at one second; PS, performance status; VC, vital capacity

**Table 4:** Patient backgrounds of lower-lobe segmentectomy cases before and after standardized inverse probability of treatment weighting

	uVATS	mVATS	p-value	SMD	IPTW weighted		
					uVATS	mVATS	SMD
n	21	33			21.7	32.8	
Age	69.2 ± 12.6	70.2 ± 6.1	0.678	0.101	69.5 ± 12.5	71.3 ± 6.1	0.183
Sex (male %)	59.1	36.4	0.097	0.454	45.5	44.5	0.020
BMI	23.8 ± 3.9	22.7 ± 3.7	0.272	0.289	23.4 ± 3.4	23.3 ± 3.8	0.028
Smoking history							
10 pack-year or more (%)	54.6	39.4	0.269	0.305	49.5	47.4	0.042
Performance status							
PS ≥1 (%)	22.7	12.1	0.298	0.280	16.0	16.0	0.000
Pulmonary function							
FEV_1_%	73.4 ± 10.3	76.8 ± 9.8	0.227	0.338	74.4 ± 8.8	76.0 ± 9.6	0.174
%FEV_1_	92.7 ± 24.3	100.3 ± 18.0	0.189	0.355	97.4 ± 26.4	98.2 ± 17.3	0.036
%VC	96.7 ± 16.9	99.3 ± 16.3	0.576	0.157	99.4 ± 18.7	98.4 ± 15.5	0.058
Charlson Comorbidity Index			0.519				
0–2	9.1	18.2		0.265	15.0	15.2	0.006
3–5	59.1	45.5		0.272	49.2	48.4	0.016
≥6	31.8	36.4		0.097	35.9	36.4	0.010
Year of surgery			0.826				
2019–2021 (%)	45.5	48.5		0.060	47.1	50.4	0.066
2022–2024 (%)	54.6	51.5		0.062	52.9	49.7	0.064
Diagnosis			0.869				
Primary lung cancer (%)	54.6	57.6		0.060	51.2	59.0	0.157
Metastatic tumor (%)	36.4	30.3		0.129	37.0	26.9	0.217
Benign (%)	9.1	12.1		0.097	11.8	14.1	0.069
Simple/complex segmentectomy							
Complex segmentectomy (%)	47.6	48.5	0.951	0.153	48.0	48.3	0.006
Experienced surgeon (≥10 cases) (%)	22.7	36.4	0.284	0.300	29.4	30.6	0.026

BMI, body mass index; IPTW, inverse probability of treatment weighting; mVATS, multiportal video-assisted thoracoscopic surgery; SMD, standardized mean differences; uVATS, uniportal video-assisted thoracoscopic surgery; %FEV_1_, percentage of forced expiratory volume at one second; PS, performance status; VC, vital capacity

### Surgical outcomes

No 30- or 90-day mortalities were observed. Intraoperative pulmonary vessel injury occurred in 12 patients, and Clavien–Dindo grade ≥III complications occurred in 10 patients. Pulmonary vessel injury was more frequent in the uVATS group than in the mVATS group (16.3% vs. 5.9%), as were grade ≥III complications (14.3% vs. 4.4%).

These complications included pleurodesis for prolonged air leak (n = 5), delayed air leak requiring drainage (n = 1), pleural effusion requiring drainage (n = 1), empyema (n = 1), cerebral infarction (n = 1), and wound dehiscence (n = 1). In the mVATS group, complications comprised pleurodesis for air leak (n = 2) and wound dehiscence (n = 1). The cerebral infarction occurred in a patient with pre-existing vascular risk factors.

Regarding the management of intraoperative bleeding, blood transfusion was required in 2 patients, and unplanned conversion to thoracotomy occurred in 3 patients. All pulmonary vessel injuries involved the pulmonary artery. Among the 12 cases, injuries occurred during vascular dissection (n = 6), stapler insertion (n = 4), excessive tension during stapler application (n=1), and slipped ligature (n = 1). Hemostasis was achieved by suture repair (n = 5), application of hemostatic agents (n=3), soft coagulation (n = 1), ligation (n = 2), or conversion to lobectomy (n = 1). Detailed data are presented in **Table 5**.

**Table 5:** Details of cause of injury and management of bleeding during uVATS and mVATS segmentectomy

	uVATS	mVATS
Cause of injury		
Dissection-related injury	5	1
Stapler insertion-related injury	3	1
Tension-related injury during stapler application	0	1
Slipped ligature	0	1
Management of bleeding		
Suture repair	3	2
Hemostat agents	2	1
Soft coagulation	1	0
Ligation	1	1
Convert to lobectomy	1	0

uVATS, uniportal video-assisted thoracoscopic surgery; mVATS, multiportal video-assisted thoracoscopic surgery

### IPTW-adjusted analysis

After IPTW, outcomes were compared between uVATS and mVATS for all segmentectomies, upper-lobe segmentectomies, and lower-lobe segmentectomies (**Table 6**). For all segmentectomies, the incidence of intraoperative pulmonary vessel injury was significantly higher in the uVATS group (22.8%) than in the mVATS group (5.5%) (p = 0.006; odds ratio [OR] 5.1, 95% confidence interval [CI]: 1.6–20.1). No other perioperative outcomes differed significantly between groups. In the upper-lobe cohort, no significant differences were observed between uVATS and mVATS for any perioperative outcomes. In contrast, in the lower-lobe cohort, the incidence of intraoperative pulmonary vessel injury was significantly higher in the uVATS group (44.8%) than in the mVATS group (2.2%) (p <0.001; OR 36.2, 95% CI: 5.0–1499.1). Grade ≥III surgical complications were also significantly more frequent in the uVATS group (18.8% vs. 0%, p = 0.005). No other outcomes differed significantly.

**Table 6:** Comparison of perioperative outcomes between uVATS and mVATS in overall, upper-lobe, and lower-lobe segmentectomy cases after IPTW

Outcome	Group	uVATS	mVATS	OR (95% CI)	p-Value
Operative time (min)	Overall	198.6 ± 79.3	195.3 ± 61.7	—	0.801
	Upper lobe	193.4 ± 68.6	196.9 ± 63.5	—	0.837
	Lower lobe	215.0 ± 78.5	190.0 ± 59.6	—	0.188
Bleeding (mL)	Overall	258.4 ± 975.2	85.0 ± 172.5	—	0.155
	Upper lobe	57.1 ± 54.5	103.1 ± 245.1	—	0.346
	Lower lobe	539.1 ± 1514.7	70.0 ± 71.8	—	0.086
Intraoperative bleeding events (%)	Overall	22.8	5.5	5.1 (1.6–20.1)	0.006
	Upper lobe	11.6	8.1	1.5 (0.26–9.1)	0.639
	Lower lobe	44.8	2.2	36.2 (5.0–1499.1)	<0.001
Postoperative complication (%)	Overall	15.7	5.3	3.3 (0.95–14.0)	0.061
(grade III or more)	Upper lobe	12.5	9.1	1.4 (0.26–7.8)	0.671
	Lower lobe	18.8	0	—	0.005
Postoperative drainage duration (days)	Overall	2.0 ± 2.2	1.8 ± 2.1	—	0.697
	Upper lobe	2.6 ± 2.4	2.1 ± 2.7	—	0.546
	Lower lobe	1.4 ± 1.4	1.2 ± 0.8	—	0.675
Postoperative hospital stay (days)	Overall	8.8 ± 8.7	7.1 ± 3.3	—	0.152
	Upper lobe	8.3 ± 3.6	7.4 ± 3.5	—	0.334
	Lower lobe	10.0 ± 13.1	6.3 ± 2.8	—	0.125

CI, confidence interval; IPTW, inverse probability of treatment weighting; mVATS, multiportal video-assisted thoracoscopic surgery; OR, odds ratio; uVATS, uniportal video-assisted thoracoscopic surgery

## Discussion

In this propensity-weighted analysis comparing uVATS and mVATS segmentectomy during the phase of institutional adoption of VATS segmentectomy, we observed that surgical safety outcomes differed according to lobar location. While no statistically significant differences were observed between uVATS and mVATS in upper-lobe segmentectomy, a higher incidence of intraoperative pulmonary vessel injury and major postoperative complications was observed in lower-lobe segmentectomy performed via uVATS. Importantly, these findings should be interpreted in the context of institutional adoption and do not indicate inherent inferiority of the uniportal approach itself.

The impact of a learning curve is an unavoidable consideration when introducing new surgical techniques. Several studies have suggested that uVATS segmentectomy requires substantial experience to achieve technical stability, with learning curve analyses indicating that approximately 38–71 cases may be required to reach procedural proficiency.^16–19)^ However, in the present study, the increased incidence of adverse events was not observed uniformly across all segmentectomies but was predominantly confined to lower-lobe resections. In contrast, upper-lobe segmentectomy did not show significantly different outcomes between uVATS and mVATS despite being performed during the same institutional period. These findings suggest a possible interaction between the learning phase and anatomical complexity, although this relationship was not formally tested and should be interpreted as hypothesis-generating. The absence of similar differences in upper-lobe procedures performed during the same period further supports this interpretation. In other words, the influence of institutional learning may not be uniform across procedures, but may be amplified in anatomically demanding settings where technical margins are narrower.

Lower-lobe segmentectomy is generally considered more technically demanding because the target vessels and bronchi are located deeper within the hilum, anatomical variations are more frequent, and intersegmental planes are often more complex.^5,6,20)^ In addition, the uniportal approach restricts visualization and instrument angulation, potentially increasing technical difficulty in these anatomically challenging settings.^21,22)^ Taken together, it is plausible that the learning phase may exert a greater impact in lower-lobe procedures, where technical margins for error are narrower. Accordingly, our findings suggest that the effect of institutional learning may be modulated by lobar anatomy rather than representing a uniform phenomenon across all segmentectomies.

These general considerations may be further explained by technical factors specific to the uniportal approach during the adoption phase. During the uVATS adoption phase, limitations in visualization and instrument angulation may lead to suboptimal exposure, making precise dissection around pulmonary vessels more technically demanding. As a result, surgeons may encounter difficulty during dissection itself, potentially increasing the risk of bleeding. Furthermore, when dissection is incomplete, additional constraints on the direction and angle of stapler insertion may arise.^22)^ In such situations, excessive tension may be applied to vascular structures during stapler placement or ligation, thereby increasing the likelihood of bleeding.

In addition, optimal exposure in uVATS relies on appropriate adjustment of lung retraction, including both the direction and magnitude of traction.^22,23)^ During the adoption phase, this adjustment may be less refined, leading to repeated repositioning or excessive grasping of the lung to achieve adequate visualization. Such repeated manipulation may contribute to parenchymal injury and may partly explain the higher incidence of postoperative air leakage observed in this setting. Although 1 patient with vascular risk factors developed cerebral infarction in our cohort, this event was considered unlikely to be directly related to a specific surgical or anatomical mechanism.

Wang et al.^21)^ reported comparable postoperative complication rates between uVATS and mVATS for complex lower-lobe segmentectomy, supporting the safety of the uniportal approach. However, their study was conducted at a high-volume center by highly experienced surgeons and did not specifically evaluate intraoperative vascular injury. Therefore, the generalizability of those findings to institutions during the introduction phase may be limited. Our results should be interpreted as reflecting early institutional experience during the adoption phase rather than as definitive evidence of comparative effectiveness.

From a practical perspective, these findings highlight the importance of careful preoperative anatomical assessment, particularly in lower-lobe segmentectomy during the adoption phase. Preoperative 3-dimensional computed tomography reconstruction may be useful for identifying vascular branching patterns and planning the line of dissection.^24)^

In addition, when adequate exposure, safe stapler angulation, or secure proximal vascular control cannot be achieved, a low threshold for modifying the surgical approach, including the addition of ports, should be maintained. Rather than defining specific segments as uniformly unsuitable, anatomical complexity should be assessed on a case-by-case basis.

Alternative approaches may also help address these technical challenges. For example, a posterior (pulmonary ligament–based) approach has been reported to facilitate access to deeply located segmental vessels, such as in dorsal basal segmentectomy, by avoiding the need for fissure dissection.^25)^ Such approaches may provide improved exposure in selected anatomically challenging cases, although they were not evaluated in the present study.

Although uVATS has been reported to offer potential benefits such as reduced postoperative pain, these outcomes were not evaluated in the present study, which specifically focused on surgical safety during the adoption phase.

Several limitations should be acknowledged.

First, this was a retrospective single-institution study with a limited sample size, which may limit generalizability.

Second, although IPTW was applied, residual confounding may have influenced the outcomes. In particular, a residual imbalance in the proportion of metastatic tumors remained in the lower-lobe subgroup (SMD >0.2), and unmeasured anatomical factors may have affected both treatment selection and outcomes.

Third, temporal changes in surgical technique may have influenced the results. During the study period, the mVATS approach evolved from a confront upside-down technique to a look-up technique. Although the year of surgery was included in the propensity score model, this adjustment may not have fully accounted for differences in visualization and operative ergonomics.

Fourth, pulmonary vessel injury was assessed retrospectively based on operative records, and blinded evaluation was not feasible, which may have introduced detection bias.

Finally, surgeon experience was limited to the early phase of adoption and may not reflect outcomes after completion of the learning curve. In addition, the choice of surgical approach was based on surgeon preference, which may have introduced residual selection bias despite statistical adjustment.

Overall, these findings suggest that during the institutional adoption phase of VATS segmentectomy, anatomical complexity may amplify the impact of the learning process, particularly in lower-lobe resections. From an implementation perspective, a stepwise case selection strategy—beginning with anatomically less demanding upper-lobe procedures and gradually progressing to complex lower-lobe resections—may represent a reasonable approach to mitigate risk during this phase. Careful procedural planning and readiness to modify the surgical approach when necessary may therefore be essential during this phase.

## Conclusions

In conclusion, during the institutional adoption phase of VATS segmentectomy, surgical safety outcomes differed according to lobar location. While upper-lobe segmentectomy showed no statistically significant differences between uVATS and mVATS, lower-lobe segmentectomy performed via uVATS was associated with a higher incidence of intraoperative pulmonary vessel injury and major postoperative complications.

These findings suggest that the impact of the learning process may be amplified in anatomically demanding lower-lobe procedures rather than representing a uniform learning curve effect. These observations should be interpreted as reflecting institutional experience during the adoption phase rather than inherent differences between surgical approaches. Careful procedural planning and readiness to modify the surgical approach may therefore be warranted during the introduction phase.

## Supplementary Material

Supplemental Table 1 Distribution of resected pulmonary segments by surgical approach and lobar location

## Declarations

### Data availability

The data that support the findings of this study are available from the corresponding author upon reasonable request.
