# Supplementary material for: Surgical Safety of Uniportal versus Multiportal VATS Segmentectomy during Institutional Adoption: Differential Impact by Lobar Location
Source: Ann Thorac Cardiovasc Surg. 2026 Jun 20;32(1):26-00042. doi: 10.5761/atcs.oa.26-00042 (PMC13293640; doi:10.5761/atcs.oa.26-00042)
Supplement: Supplemental Table 1 — Distribution of resected pulmonary segments by surgical approach and lobar location [file atcs-32-1-26-00042-s001.pdf]

Supplemental Table 1. Distribution of resected pulmonary segments by surgical approach and lobar location

Surgery types	uVATS (n = 49)	mVATS (n = 68)
Right upper lobe	10	11
S1	2	4
S1+S2		2
S1a+S2	2	
S1b		1
S2	3	2
S3	3	2
Right lower-lobe	12	19
S6	8	7
Basal segment	1	2
S7+S8		2
S7+S8+S9	1	
S7a+S8	1	
S8		4
S6+S9+S10		1
S9+S10	1	3
Left upper-lobe	16	24
Upper segment	4	10
Lingular segment	1	3
S1+2	7	5
S1+2a+b		1
S3	4	2
S1+2a+S3		1
S3+S4+S5		1
S3a+S1+2c+S4+S5		1
Left lower-lobe	11	14
S6	2	6
Basal segment	2	2
S6+S10	1	
S8	1	2
S8+S9		1
S9	1	
S9+S10	4	1
S10		2
